# Winter Nights during Summer Time: Stress Physiological Response to Ice and the Facilitation of Freezing Cytorrhysis by Elastic Cell Wall Components in the Leaves of a Nival Species

**DOI:** 10.3390/ijms21197042

**Published:** 2020-09-24

**Authors:** Matthias Stegner, Barbara Lackner, Tanja Schäfernolte, Othmar Buchner, Nannan Xiao, Notburga Gierlinger, Andreas Holzinger, Gilbert Neuner

**Affiliations:** 1 Department of Botany, University of Innsbruck, 6020 Innsbruck, Austria; barbara.lackner@hotmail.com (B.L.); tanja.schaefernolte@t-online.de (T.S.); andreas.holzinger@uibk.ac.at (A.H.); gilbert.neuner@uibk.ac.at (G.N.); 2 Department of Biosciences, University of Salzburg, 5020 Salzburg, Austria; mail@o.buchner.co.uk; 3 Institute for Biophysics, University of Natural Resources and Life Sciences (BOKU), 1190 Vienna, Austria; nannan.xiao@boku.ac.at (N.X.); burgi.gierlinger@boku.ac.at (N.G.)

**Keywords:** alpine plants, cold hardiness, freeze dehydration, ice nucleation, ice management, low temperature, radiative cooling, *Ranunculus glacialis*

## Abstract

*Ranunculus glacialis* grows and reproduces successfully, although the snow-free time period is short (2–3 months) and night frosts are frequent. At a nival site (3185 m a.s.l.), we disentangled the interplay between the atmospheric temperature, leaf temperatures, and leaf freezing frequency to assess the actual strain. For a comprehensive understanding, the freezing behavior from the whole plant to the leaf and cellular level and its physiological after-effects as well as cell wall chemistry were studied. The atmospheric temperatures did not mirror the leaf temperatures, which could be 9.3 °C lower. Leaf freezing occurred even when the air temperature was above 0 °C. Ice nucleation at on average −2.6 °C started usually independently in each leaf, as the shoot is deep-seated in unfrozen soil. All the mesophyll cells were subjected to freezing cytorrhysis. Huge ice masses formed in the intercellular spaces of the spongy parenchyma. After thawing, photosynthesis was unaffected regardless of whether ice had formed. The cell walls were pectin-rich and triglycerides occurred, particularly in the spongy parenchyma. At high elevations, atmospheric temperatures fail to predict plant freezing. Shoot burial prevents ice spreading, specific tissue architecture enables ice management, and the flexibility of cell walls allows recurrent freezing cytorrhysis. The peculiar patterning of triglycerides close to ice rewards further investigation.

## 1. Introduction

In the nival life zone, the snow-free time slot is short, not exceeding 2–3 months [1,2]. During this time, freezing temperatures are frequent. Despite global warming, frost is anticipated to remain a predominant factor, as alpine plants seem to be exposed to an unchanged frost risk due to the earlier loss of snow cover [3]. At 3450 m a.s.l. on Mt. Brunnenkogel (Pitztal Glacier) in a canopy of *Ranunculus glacialis*, night frosts were recorded on 68% of 74 snow-free nights [4]. After such nights, plants can be found stiffly frozen in the morning. At these nival elevations in the Tyrolean Alps, free-air temperatures can drop down to −15.6 °C during cold snaps from June to August [4,5,6,7]. However, weather station data fail to capture the life conditions of small statured alpine plants, such as herbs and grasses [8]. For subalpine environments, an energy balance analysis of nocturnal leaf temperature revealed that leaves can even be 3–8 °C colder than the air [9]. In the nival life zone, the environmental forces driving the leaf temperature below air temperature may be even stronger. However, so far the leaf temperatures of nival plants close to their upper elevational distribution range have not been recorded, although it is inevitably necessary to gain insight into frost survival.

*R. glacialis* is one of the nival herbs of the European Alps that can grow at the highest elevations (e.g., at 4275 m a.s.l. in the Swiss Alps [10]). The species is ice-tolerant throughout the active growing period and is one of the most freezing-resistant herbs in the European Alps [11]. In earlier studies, the median lethal temperature (LT_50_) of *R. glacialis* leaves from lower elevations (2600–2800 m a.s.l.) ranged between −8.2 °C and −9.4 °C [5,7,12]. In a recent survey, stronger environmental demands at 3200 m a.s.l. pushed the freezing resistance of leaves of *R. glacialis* down to −13.0 °C (LT_50_) [11]. In contrast to many other alpine plant species, not only the leaves but also the reproductive parts are ice-tolerant, and even during anthesis flowers survive down to −8 °C (LT_50_; [7]). Little is known about how whole plants freeze during natural (radiative) night frosts. Particularly for high-elevation growing sites, there is no record at which temperature and how frequently leaves are subjected to ice nucleation and extracellular ice formation, or if they manage to stay unfrozen and for how long.

Night frosts and ice formation in leaves sensitively affect photosynthetic performance. While ice in the intercellular space per se inhibits CO_2_ gas diffusion and photosynthesis, also the after-effects of leaf freezing are well known. Depending on their severity, freezing temperatures and ice formation in leaves can implicate significant photosynthetic depressions for several consecutive days [13]; on the other hand, frozen leaves of afroalpine giant rosette plants regained their full photosynthetic performance immediately after thawing [14]. Given the high frequency of night frosts at high elevation, it can be questioned whether a regular photosynthetic performance in leaves of *R. glacialis* is possible and how the challenge of frequent night frosts is managed at a physiological level.

Once ice forms in ice-tolerant plant tissues, cells are exposed to mechanical stress by extracellular ice formation and freeze dehydration. These processes can result in the deformation (“caving-in”) of cell walls, so-called freezing cytorrhysis [15,16]. Structural changes in cells during freezing cytorrhysis were demonstrated in cryo-microscopic studies (e.g., *Pachysandra terminalis* [17]; *Sphagnum capillifolium* [18]). Freezing cytorrhysis is, down to critical temperature thresholds, a fully reversible process, as melting water from extracellular ice re-enters into the cells during thaw, causing their return to normal shape [19]. A prerequisite for cell shape changes may be a highly elastic and deformable cell wall. So far, neither the structure nor the chemical composition of cell walls enabling freezing cytorrhysis have been studied. In a recent study of freezing tolerance in a streptophytic green alga, even cell wall reinforcements were established as a consequence of the freezing stress [20]. Furthermore, it is not known how the plasma membrane behaves during freezing cytorrhysis in intact plant cells, as the few available studies were conducted on isolated protoplasts [21,22]. Still, structural and functional impacts on the plasma membrane and reactive oxygen species (ROS)-induced damage are currently implicated as the main two types of freeze-thaw injury lesions (for a recent review, see Arora [19]). Although undoubtedly observed, the overall process of freezing cytorrhysis, the extent and the temporal- and temperature-dependent dynamic of cellular shrinkage, alterations in the plasma membrane, and the cell wall prerequisites are not much understood.

At a high-elevation growing site (3185 m a.s.l., Austria) of *R. glacialis*, we aimed to assess: (1) How frequently ice forms in the leaves within the short snow-free period and how whole plants freeze under natural (radiative) night frosts. Freezing exotherms and whole plant freezing were monitored on site and compared to the results of infrared differential thermal analysis (IDTA) obtained under controlled freezing conditions. (2) The effect of ice formation on the “after-freezing physiological performance” was determined by measurements of the chlorophyll fluorescence and photosynthetic gas exchange. (3) In frozen leaves, ice masses were localized with cryo-microscopy in reflected polarized light (CM_rpl_; [23]), and the temporal- and temperature-dependent dynamic and the extent of freezing cytorrhysis were measured by cryo-microscopy. (4) Plasma membrane staining with the amphiphilic styryl dye FM 1-43 was additionally employed to monitor changes in the plasma membrane during freezing cytorrhysis. (5) To gain first insight into the peculiar cell wall composition and cytosolic components enabling the remarkable shape changes during freezing cytorrhysis, we applied Raman imaging.

## 2. Results

### 2.1. Leaf Temperature and Leaf Freezing in the Field

**Figure 1:**
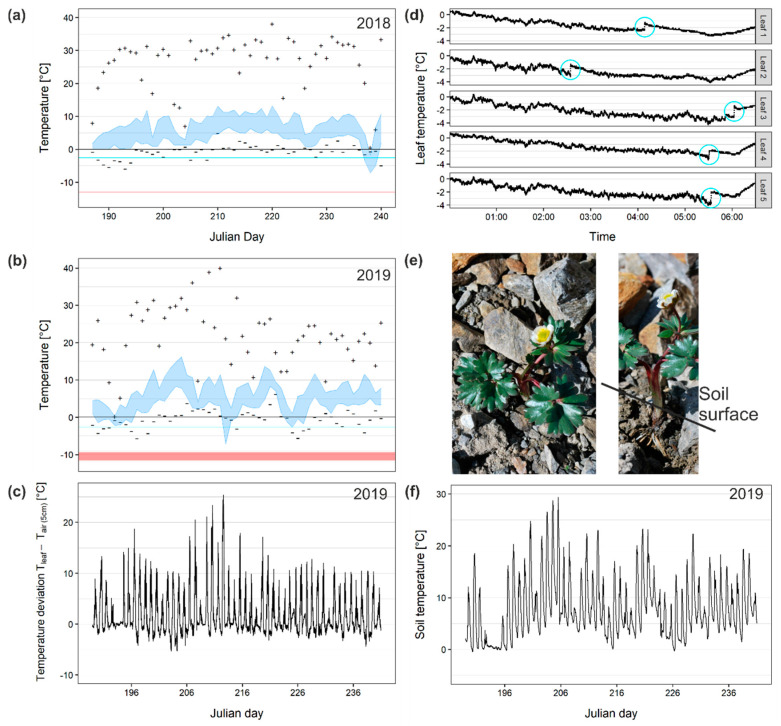
In situ leaf temperatures of *R. glacialis* at 3185 m a.s.l. compared to the free air temperature in the growing periods of 2018 and 2019: (**a**,**b**) The air temperature at 2 m (blue ribbon) had a narrow daily amplitude compared to the amplitude of leaf temperature (+ signs indicate the daily maximum and—signs the daily minimum). Nocturnal leaf temperature minima were mostly lower than the 2 m air temperature minima. (**c**) Comparison between the air temperature at the average canopy height and the leaf temperatures showed a temperature depression of leaves of up to −5.7 °C in 2019. Still, the minimum temperatures of the leaves never dropped below the median lethal temperature (LT_50_) values (red line/ribbon in (**a**,**b**)). (**d**) In situ freezing exotherms occurred at −2.6 ± 0.9 °C (turquoise line in (**a**,**b**)). The temporal leaf freezing behavior of 5 leaves of one *R. glacialis* individual over the course of a single night is exemplarily shown and indicates that single leaves froze at different times (turquoise circles). Air temperature (2 m) did not drop below 0.0 °C during that night. (**e**) The leaf petioles of *R. glacialis* leaves are connected to the shoot approx. 5 cm below the soil surface. (**f**) Monitoring the soil temperature in a comparable depth revealed that the temperatures did not drop severely below 0 °C throughout summer. The warm soil may act as a thermal barrier on many nights, preventing ice propagation from one leaf into another and, by this, promoting supercooling at the leaf level.

Indeed, the in situ leaf temperatures deviated greatly from the air temperature; the daily minimum and maximum air temperatures were in a narrow range between −7.2 and +16.2 °C within the growth periods of 2018 and 2019 (blue ribbon in Figure 1a,b). The daily leaf temperature maxima (+signs in Figure 1a,b) occasionally even reached +40 °C, and the daily leaf temperature minima (−signs in Figure 1a,b) were in extreme −5.9/−6.0 °C (2018/2019). The leaf temperature minima mostly (92% ± 2%) were lower than the air temperature minima. Exceptional cases were after snowfalls, when leaves were thermally insulated by snow. In these situations, a constant leaf temperature of around 0 °C was recorded, whereas the air temperature often was lower. Nocturnal leaf temperatures dropped below 0 °C at a rate of 50% ± 9% (2018) and 62% ± 4% (2019), respectively, while air frosts occurred only at rates of 7% and 26%. When the air temperature was above 0 °C, the leaves could already be exposed to freezing conditions. The largest leaf temperature depression compared to the 2 m air temperature was −9.3 °C. Even a comparison between the leaf temperature and air temperature at the plant canopy level showed a nocturnal temperature depression of the leaves of up to −5.7 °C (Figure 1c). The daily mean leaf temperatures were mostly close to the air temperature maxima (data not shown).

In situ freezing exotherms were detected in leaves at −2.6 ± 0.9 °C (*n* = 77) (turquoise lines in Figure 1a,b). Leaf freezing events were detected between 09:20 p.m. and 06:03 a.m.; freezing occurred on average at 02:10 am (SD = 153 min). Leaves in which ice formation events had been detected endured unfrozen for 164 ± 83 min before ice nucleation. After the ice nucleation, they stayed frozen for 294 ± 168 min.

We analyzed the temporal freezing behavior of individual plants during the course of cold nights. Exemplarily, in Figure 1d the leaf temperatures from five leaves of one individual plant are shown. The freezing exotherms did not happen at the same time. The timespans between the single-leaf freezing events were up to several hours. Thus, leaves froze independently. Ice from one leaf did not spread into the other leaves immediately, suggesting a barrier between the individual leaves. However, the leaves are connected via the shoot tissue, which is buried several centimeters below the ground (Figure 1e). The soil temperature in a depth where the shoot is located was in most nights (89%) warmer than 0 °C (Figure 1f). The absolute soil temperature minimum was −0.5 °C.

Neither in 2018 nor in 2019 did leaf temperatures reach lethal temperatures. The median lethal temperature (LT_50_) values were assessed four times in the growing seasons of 2018 and 2019, and were between −9.0 °C and −13.0 °C (red line/ribbon in Figure 1a,b).

The spatio-temporal freezing behavior observed in the field was corroborated by IDTA measurements on potted individuals of *R. glacialis* exposed to controlled freezing down to −14 °C in the lab. Similar to the observation in the field, the leaves froze independently and one after another (Supplementary Figure S1a–g; Supplementary Video S2). Ice nucleation did not start at a distinct point on the leaf blade. As the mesophyll froze, the leaf blade at first exhibited a brindled pattern (Figure S1d), which transitioned into a more homogenous pattern (Figure S1e). Only one freezing event was detected in every leaf, even if leaves were frozen to a temperature of −14 °C, which is below the LT_50_.

### 2.2. After-Effects of Extracellular Ice Formation on Photosynthesis

**Figure 2:**
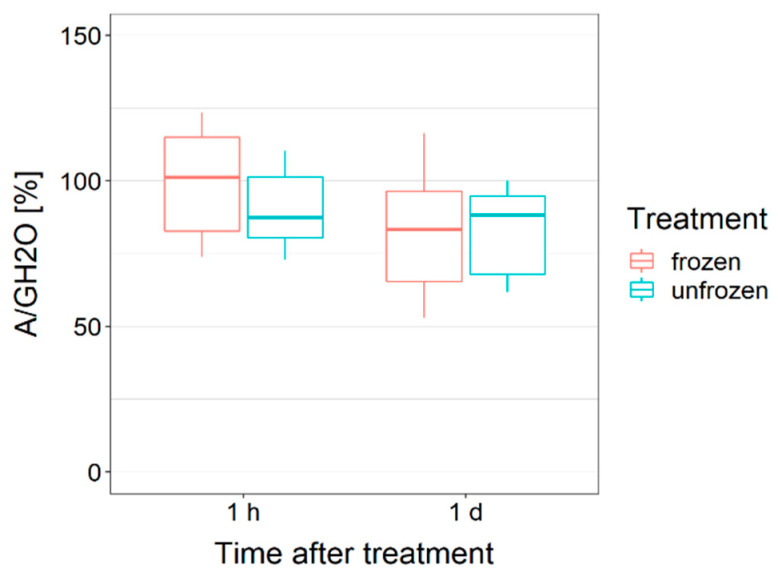
After controlled exposure to subzero temperatures, the photosynthetic gas exchange of detached leaves was not different, irrespective of whether the leaves were artificially ice nucleated or not. In a laboratory freezer, the leaves were ice nucleated and kept frozen at −3 °C for 4 h (red) or remained unfrozen (no ice formation within the mesophyll) and exposed to −1 °C for 4 h (blue). To minimize the effects of different stomata widths and natural differences between the leaves, the net assimilation rate (A) of each single leaf was related to the diffusive conductance (GH2O) and then to its initial value before the cold treatment. Box-plots show median, 25th and 75th percentile, maxima and minima.

Under the experimental conditions, neither 1 h after the treatments (frozen at −3 °C vs. unfrozen at −1 °C) nor 1 day afterwards did the detached leaves show a significant difference in gas exchange (net assimilation rate divided by the diffusive conductance; A/GH2O) (Figure 2). A/GH2O is referred to the control values before the treatment. Additionally, the dark respiration rate when related to the leaf diffusive conductance (R_d_/GH2O) was unaffected by the freezing treatment and time. These findings additionally apply to the efficiency of PS II (Fv/Fm). However, measurements revealed that the treatment (frozen vs. unfrozen) was almost significantly influencing Fv/Fm (*p* = 0.050). Before the treatments, the Fv/Fm was 0.79/0.79 (unfrozen/frozen), 1 h after the treatments it was 0.79/0.76, and 1 day after it was 0.77/0.75. The overall Fv/Fm values were not significantly different; consequently, ice in the leaves of *R. glacialis* does not affect the photosystem II efficiency.

### 2.3. Ice Management—Accommodation of Ice Masses in the Leaves of R. Glacialis

**Figure 3:**
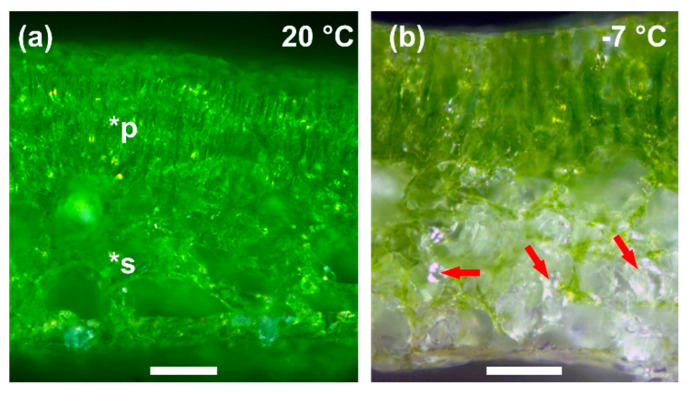
Microscopic images of the cut surface of leaves of *R. glacialis*. Images were either taken from cut leaves that were (**a**) unfrozen at +20 °C or (**b**) had been ice nucleated at −2 °C and were frozen to a sublethal temperature of −7 °C. In the upper part of the images, tightly packed cells with a cylindrical shape—i.e., palisade parenchyma tissue (*p)—can be seen. The spongy parenchyma tissue (*s) in the lower part accommodates huge intercellular spaces between heterogeneously shaped cells. In (**b**), by the use of crossed polarization filters large ice crystals (red arrows) can be identified. The ice crystals fully occupied but additionally seemed to widen up the intercellular spaces. In contrast, within the palisade tissue no major ice masses could be seen. Bar width: 100 µm.

Cryo-microscopy in reflected polarized light (CM_rpl_) revealed that, particularly in the spongy parenchyma, huge ice masses are accommodated (Figure 3).

Leaves of *R. glacialis* are bifacial with adaxial palisade layers and an abaxial spongy parenchyma tissue (Figure 3a). Investigations on cross sections revealed a total thickness of the palisade tissue layers of 278 ± 36 µm (*n* = 30) and spongy tissue layers with a thickness of 309 ± 67 µm (*n* = 30). The palisade cells are tightly packed and close together, with apparently only narrow intercellular spaces. Cells in the spongy parenchyma tissue are heterogeneously shaped and have much larger intercellular spaces than the palisade cells. The intercellular spaces occupied 13% ± 3% (*n* = 3) of the total area of the leaf cross sections. With CM_rpl_ in frozen leaves, no major ice masses could be detected in the palisade tissue. Ice masses were exclusively found in the intercellular spaces of the spongy parenchyma tissue (Figure 3b). Ice crystals not only seem to occupy, but even to actually widen up the intercellular spaces.

### 2.4. Freezing Cytorrhysis

**Figure 4:**
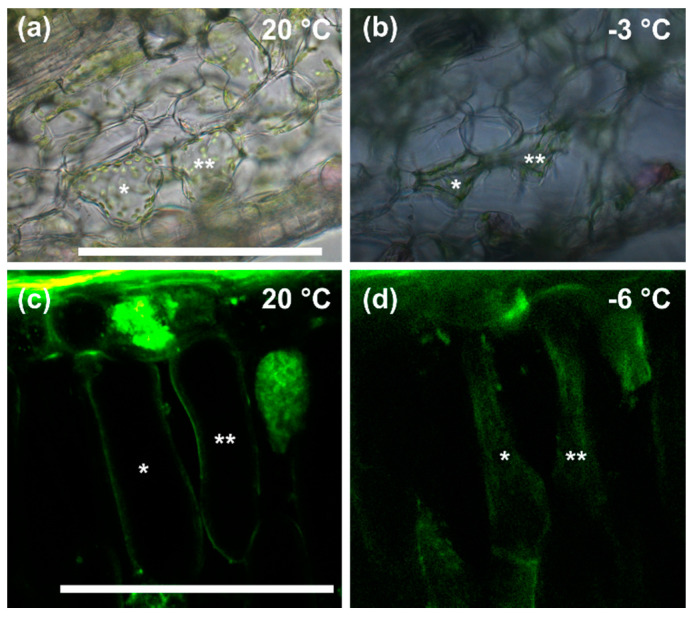
Mesophyll cells of *R. glacialis* responded to extracellular ice by severe freezing cytorrhysis: Light microscopic images of spongy parenchyma cells in leaf cross sections of *R. glacialis* (**a**) at +20 °C and (**b**) after ice nucleation during freezing at −3 °C. (**c**) Confocal laser scanning images of palisade parenchyma cells stained with FM 1-43 at +20 °C (excitation 488nm; emission 505–550 nm)—note the clear visibility of the plasma membrane as a sharp line (asterisks)—and (**d**) during freezing at −6 °C, note the blurred appearance of the stained inward-facing plasma membrane. Spongy parenchyma (**b**) as well as palisade parenchyma cells (**d**) (indicated by asterisks) underwent severe freezing cytorrhysis. Bar width: 100 µm.

By the controlled freezing of cross sections of leaves of *R. glacialis* viewed by light- and confocal laser scanning microscopy, the specific response of the palisade and spongy parenchyma cells to the presence of extracellular ice could be monitored. At +20 °C, the spongy parenchyma cells were fully turgescent (Figure 4a). Ice crystal formation occurred in the light microscope-temperature-controlled chamber (LM-TCC, [24]), usually at freezing temperatures between −1.5 and −2.0 °C. Below these temperatures, the onset of the freezing cytorrhysis of cells could be observed in the bright-field image by an irregular cell shape (Figure 4b). The staining of the palisade parenchyma cells with FM 1-43 resulted in a straight line representing the plasma membrane in +20 °C control cells (Figure 4c), whereas cells frozen at −6 °C showed a cytorrhyzed appearance, FM 1-43 stained the inward facing plasma membranes, and also a faint staining was detected in the cell lumen (Figure 4d).

**Figure 5:**
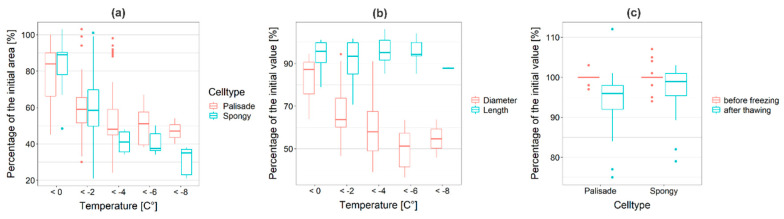
(**a**) Cross sections of *R. glacialis* were exposed to controlled freezing and extracellular ice. The cell area (CA) of mesophyll cells was successively reduced with decreasing temperatures. Below −3 °C, the CA of the palisade parenchyma cells (red; *n* = 25) was reduced to 54% ± 15%, whereas the CA of the spongy parenchyma cells (blue; *n* = 15) was reduced to 48% ± 15%; n.s. difference. (**b**) The cell diameter (red) and the cell length (blue) of the palisade parenchyma cells responded biased to the presence of ice. While the cell diameter was severely reduced, the length of the cells remained nearly unchanged. (**c**) After thawing (blue), the cells increased towards their initial CA before freezing (red). Box-plots show median, 25th and 75th percentile, maxima, minima and outliers (dots).

Once ice nucleation occurred, freezing cytorrhysis could be immediately observed and became more severe with decreasing temperatures (to −8 °C; Figure 5a), suggesting structural prerequisites allowing the freeze dehydration and caving-in of cells as a physical response to extracellular ice. Below −3 °C, the cell area (CA) of the palisade cells (*n* = 25) was reduced to 54% ± 15% of the initial value, whereas the spongy parenchyma CA (*n* = 15) was reduced to 48% ± 15% of the untreated controls; the CA of the spongy parenchyma cells was more pronouncedly reduced than that of the palisade cells, but not significantly (*p* = 0.06). Upon extracellular freezing, the palisade cells showed an intensive reduction in diameter, but not in cell length (Figure 5b). After thawing, the CA and cell dimensions increased towards their initial values (Figure 5c).

### 2.5. Chemical Imaging of the Cell Wall and Cell Components

**Figure 6:**
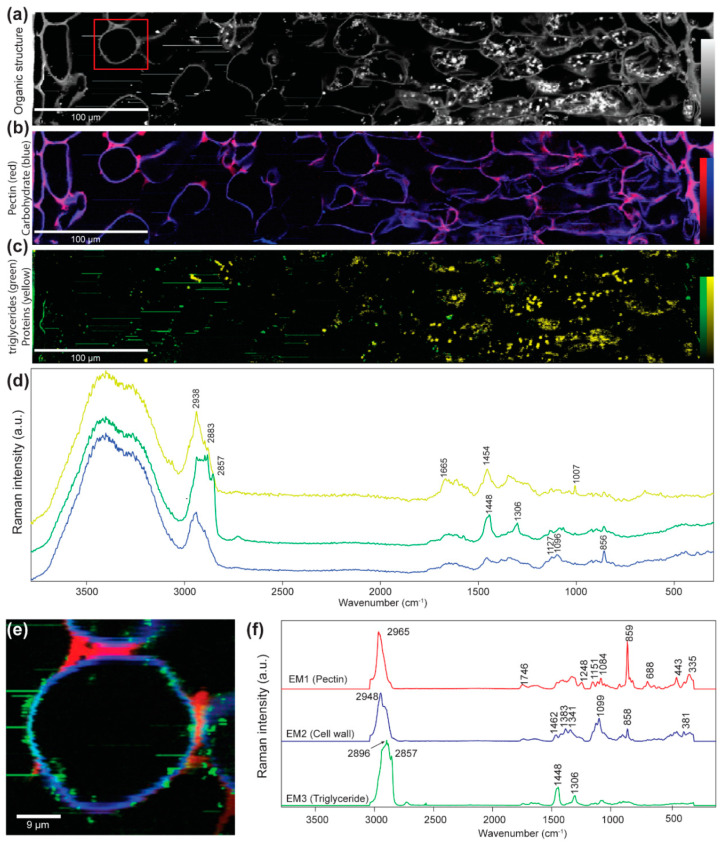
Raman imaging of a leaf cross section of *R. glacialis*: (**a**) Visualization of all organic structures by integrating the CH-stretching region from 2831 to 3009 cm^−1^ reveals the spongy (left side) and palisade parenchyma (right side) and their cell contents. (**b**) Imaging the cell wall based on the integration of carbohydrate bands from 1016 to 1184 cm^−1^ in blue and highlighting the pectin accumulation by the 856 cm^−1^ band integration between the cells in red and pink (overlay of both integration images). (**c**) In the spongy parenchyma, triglycerides are visualized by integrating the 2857 cm^−1^ band (green), while the palisades reveal a high protein accumulation by integrating the 1660 cm^−1^ amide band (yellow). (**d**) Average spectra extracted from regions rich in carbohydrates (blue) (based on Figure S3b, blue area), triglycerides (green) (extracted from Figure S3c, green area), and proteins (yellow) (extracted from Figure S3c, yellow area), respectively. (**e**) A zoom into the spongy parenchyma based on non-negative matrix factorization (NMF) highlights the pectin accumulation (red) and lipid droplet lining (green) of the cell wall (blue). (**e**,**f**) The endmember spectra confirm pure pectin between the cells (EM1, red), a pectin-rich cell wall (EM2, blue), and the triglyceride nature of the lipid droplets (EM3, green) (see also Supplementary Figure S3a–c).

Based on the band integration of marker bands for carbohydrates, pectin, lipids, and proteins, insights into the cell wall composition as well as the lumen components were gained (Figure 6a–c). Integrating the CH-stretching shows all organic structures [25], with the cell walls with medium intensity in grey and the components inside the lumen with the highest intensity in white (Figure 6a). Restricting the band integration from 1016 to 1184 cm^−1^ visualizes all the cell walls based on carbohydrate bands [26] (Figure 6b, blue). Extracting an average spectrum from the regions highlighted in blue confirms the carbohydrate nature of the cell wall (Figure 6d, blue spectrum). The carbohydrate bands at 1127 and 1095 cm^−1^ are clearly visible, while the cellulose marker band at 380 cm^−1^ is too weak for image generation. In contrast, the characteristic pectin band at 856 cm^−1^ [27] represents the strongest band within the cell wall spectrum (Figure 6d, blue spectrum). Integrating this pectin marker band reveals the highest intensity in the apoplast between the cells (Figure 6b, red and pink color). Integrating the protein and lipid marker bands at 2857 and 1660 cm^−1^, respectively, shows a very protein-rich palisade parenchyma, whereas in the spongy part more lipids accumulate as small vesicles along the cell wall (Figure 6c, green lipids, yellow proteins). The average spectrum referring to the yellow regions confirms the protein nature of these components by protein bands at, e.g., 1660 and 1007 cm^−1^ (Figure 6d, yellow spectrum), which have been assigned to the amide I and phenylalanine vibrations in proteins [28]. The extracted average spectrum of the highlighted green lipid-rich regions shows bands at 2857 and 2883, 1448, and 1306 cm^−1^ (Figure 6d, green spectrum), which can be assigned to the CH-stretching, CH-bending, and CH-wagging of triglycerides [29]. A zoom into the spongy parenchyma based on non-negative matrix factorization (NMF) analysis gives, indeed, an endmember clearly representing pectin with the marker band at 856 cm^−1^ and a high accumulation between the cells (Figure 6e,f, red). The cell wall itself is based on carbohydrates (blue) and tiny lipid droplets (green) accumulating along the cell wall (Figure 6e,f). Detailed analysis of the endmember pectin spectrum (Figure 6f, EM1, red) shows a strong match with a pectin 55–70% esterified (Supplementary Figure S3a), whereas in the endmember cell wall spectrum (Figure 6f, EM2, blue) a carbohydrate mixture of pectins and cellulose fits best and confirms the high pectin content of the cell wall (Supplementary Figure S3b). The lipid endmember spectrum (Figure 6f, EM3, green) with high CH-stretching bands at 2986 and 2857 cm^−1^ and characteristic bands at 1448 and 1306 cm^−1^ points to a triglyceride such as tripalmitin, which shows exactly the same marker bands (Supplementary Figure S3c) [29]. Lipid droplets (Figure 6e, green) accumulate along the cell wall.

## 3. Discussion

In the nival habitat, only a short snow-free time slot is available for plant growth; the investigation site was not covered by snow on 82 days in 2019. Therein, recurrent night frosts at the plant level pose a particular challenge. Our results show that it is not so much the severity of the night frosts but rather their high frequency of up to 62%. The absolute leaf temperature minima were milder than those of air, as during cold spells with very low air temperature minima the plants were covered by a protective snow layer. Cold air temperatures did not necessarily mean cold leaf temperatures and vice versa. Overall, as suggested earlier [8], our results confirm that it is not possible to draw any conclusions for the leaf temperature minima of small statured plants from standard meteorological data, particularly in the nival zone. Neither the 2 m air temperature nor the air temperature at the average canopy height are reliable indicators for plant temperatures. The measured leaf temperature depressions may arise under certain environmental conditions, such as low wind speed and a clear night sky (low cloudiness) [9,30]. Such conditions may be favored especially at higher elevations due to a lower moisture content and lower atmospheric density [9]. The temperature depressions of *R. glacialis* were in the range measured at 3230 m a.s.l. in a subalpine environment of the Rocky Mountains by Jordan and Smith [9]. At lower elevations, the nocturnal temperature depressions were particularly lower (grasses −3.7 °C [30], *Eucalyptus* −1–−3 °C [31], cited in Jordan and Smith [9]) supporting the hypothesis that the temperature depressions of leaves may rise with the increasing elevation of the growing site.

At 3185 m a.s.l., the leaves of *R. glacialis* were frequently (50% ± 9% (2018) and 62% ± 4% (2019)) exposed to nocturnal freezing temperatures. In contrast, during the unusually warm summer of 2018, the air temperature dropped only once, during a cold snap with snowfalls, severely below 0 °C. When the leaf temperature drops below 0 °C, two scenarios are possible: either the leaf tissues remain unfrozen or upon ice nucleation extracellular ice forms. Our results show that, in the nival habitat, ice nucleation occurred in the leaves of *R. glacialis* at −2.6 ± 0.9 °C. This corresponds to the ice nucleation temperatures measured in nature in various species in different environments (−1.9 °C in apple [32], between −1.2 and −2.1 °C in temperate woody species [33], −3.3 °C in *Pinus cembra* leaves [34], between −1.5 and −2.5 °C in grasses [35,36]).

In the field as well as in the lab experiments, individuals of *R. glacialis* never froze at once. Independent ice nucleation events were necessary. Ice propagation from one leaf into another is thermally prevented, as the connecting shoot is buried at a depth of several centimeters in the unfrozen soil. Already, Körner [37] suggested that, for most alpine plants, the burial of vegetative shoot apices, basal leaf meristems, and premature reproductive organs several centimeters below the soil surface is a strategy to protect them from deleterious freezing temperatures (morphological avoidance). Our results indicate that this morphology even prevents ice spreading between individual leaves—i.e., the freezing avoidance of above-ground plant parts. This way, single leaves of *R. glacialis* can remain unfrozen during night frosts. Staying unfrozen as long as possible may be assigned to a sort of transient ice avoidance strategy as a first line of defense. In case of ice nucleation, a second line of defense by reversible freezing cytorrhysis has to be assembled. To minimize the duration of exposure to the peculiar strains upon freezing cytorrhysis, transient ice avoidance may be supportive. Still, the effects of low temperature exposure on photosynthesis were similarly independent from the kind of freezing process, neither unfrozen nor extracellular ice formation and freezing cytorrhysis had a specific negative impact on the photosynthetic gas exchange and photosystem II functioning. These results corroborate earlier findings from an afroalpine plant [14].

The minimum leaf temperatures during the observation periods in 2018 and 2019 never dropped below the maximum freezing resistance capacity; hence, the leaves of *R. glacialis* are well adapted to the low-temperature conditions of the nival zone. Exhibiting such a high freezing resistance even during the growing period [11,37] in the presence of high water contents and turgidity sheds light on the importance of their enormous genetic potential to endure cold-temperature conditions.

The tremendous cellular changes observed upon ice nucleation and freezing cytorrhysis accompanied by severe freeze dehydration may emphasize the importance of the ice avoidance strategy. However, the accommodation of ice masses and enduring the resulting strains is key for survival and reproduction in the nival zone [5,7]. Cryo-microscopy in reflected polarized light revealed that the majority of extracellular ice forms in the intercellular spaces of the spongy tissue; no ice masses were visible between the palisade cells. How spaces for ice accommodation account for species-specific frost survival to puffer ice masses and alleviate freezing damage has, with some exceptions [38,39], hardly been addressed. Especially for plants, enduring severe freezing events during the growing period with high leaf-water contents, such spaces may be crucial. Considering the phase transition volume increase from water to ice of approx. 9% [40], the intercellular spaces in the spongy tissue might act as a predetermined space for ice accommodation. Consequently, extracellular ice causes a steep water potential gradient, resulting in the temperature-dependent withdrawal of cellular water to the extracellular ice bulk [41,42].

At the cellular level, severe freezing cytorrhysis was detected in the palisade cells (mean values decreased to 54% of the initial CA) as well as the spongy parenchyma cells (mean values decreased to 48% of the initial CA). In comparison, the peat moss *Sphagnum capillifolium* showed a cell diameter decrease down to 43% of the initial value during freezing cytorrhysis at −5 °C—i.e., an approximated volume reduction of 82% [18]. The dehydration-induced volume reduction was about 80% in ivy leaves measured at −10 °C with NMR spectroscopy by Hansen and Beck [43]. As the spongy parenchyma cells of *R. glacialis* are very irregularly shaped, quantifying the decrease in cell volume is difficult. For the palisade cells, by approximating a cylindrical shape the volume decrease would be 73%. Indeed, despite morphologic and phylogenetic differences, the quantity of freezing-induced dehydration seems quite similar among the three species.

As illustrated by the FM 1-43 staining of palisade cells, mostly the cell diameter was reduced, but not the cell length. This may suggest structural differences within the cell wall of palisade parenchyma cells, or else that the tissue architecture of the tightly packed palisade cells only allows changes in diameter.

Whether the plasma membranes change during freezing is more difficult to judge even when stained by FM 1-43. A study by Yamazaki, Kawamura and Uemura [22] has shown that the plasma membrane of isolated protoplasts undergoes characteristic changes upon extracellular freezing—i.e., a reduction in the surface area is counteracted by the formation of freeze-induced vesicles (FIVs) [22]. The staining of the plasma membrane of palisade cells of *R. glacialis* by FM 1-43 clearly allowed to determine the changes between the untreated and the frozen cells. Although diffuse changes in a blurred plasma membrane were captured during freezing, the distinct formation of FIVs could not be demonstrated, but their existence cannot be excluded.

The fact that, particularly in the intercellular areas of the spongy parenchyma, large masses of ice crystals were observed by CM_rpl_ nicely correlates with the observation of an even stronger freezing cytorrhysis within this tissue, which, however, was not significant. The freeze dehydration of palisade parenchyma cells must occur via the spongy parenchyma cells. It seems to be the cell walls of the spongy parenchyma cells where the intracellular water is transferred to the extracellular ice. This indicates that the determination of where ice forms may be decided by predetermined local cell wall patches through which water is transferred from the supercooled cells to the apoplast. Additionally, this might explain why, immediately after thawing, the CA of the spongy cells was higher than that of the palisade cells (Figure 5c); this might indicate a delay in rehydration due to the greater distance to the melted ice masses.

Raman spectroscopy demonstrates peculiar molecular components in the cell walls but also in the lumen of cells of the mesophyll of *R. glacialis*. Most striking is the pectin richness of the cell walls, with a relatively high esterification rate (55–70%), and the lipid droplets (triglyceride) prominently occurring in the spongy tissue. The ice barrier tissue below buds of Norway spruce that transfers water from the bud to the extraorgan ice masses in the shoot below was also characterized by pectin-rich cell walls [44,45]. Pectin accumulation in the cell wall may aid the shifting of liquid water to the ice masses in the apoplast. At the same time, the pectin in cell walls may block ice penetration in the opposite direction. Recent studies have revealed that the accumulation of triglyceride plays an important role in abiotic stress response, especially in plant freezing tolerance [46,47,48]. The localization of triglyceride in the spongy tissue, where the major ice masses are accommodated, may be related to freezing stress response, and highlights the interaction between the tissue architecture and function.

## 4. Materials and Methods

### 4.1. Study Site and Plant Material

*Ranunculus glacialis* L. was studied at 3185 m a.s.l. at Mt. Kleiner Isidor (N 46°58.424 E 11°6.436 E) in the Austrian Central Alps. The site is located at the upper distributional vertical range of the species—i.e., 2300 to 3200 m a.s.l.—but individuals can occasionally be found at even higher elevations [49]. *Ranunculus glacialis* predominantly grows on limestone-free and nutrient-poor soils, mostly on moraines or screes [50].

Samples for the investigations were taken either from Mt. Kleiner Isidor (in 2018 and 2019) or, as in 2011, from the location Eisgrat (N 46°59.267 E 11°6.983, 2850 m a.s.l.). In 2011, individuals were excavated and potted in a substrate mixture (2:1) of nutrient-poor and limestone-free soil and Vermiculite. Potted individuals were transferred to the Alpine Garden of the University of Innsbruck (N 47°12.66 E 11°27.07, 1950 m a.s.l.) on Mt. Patscherkofel. From these cultivated plants leaf samples could be collected for cryo-microscopic investigations. In 2018 and 2019, whole individuals were dug out and potted immediately on site into natural soil. Plants were transported in a chilled thermal bag to the lab. For the Infrared Differential Thermal Analysis (IDTA), they were stored in darkness at +4 °C until the beginning of the experiments. For the gas exchange measurements, the potted plants were transferred into a growth chamber (8 h night: 0 °C; 16 h day 20 °C) for 3 days to eliminate potential physiological limitations due to prior frost nights. All the other experiments (determination of freezing resistance, cryo-microscopy in reflected polarized light, and Raman imaging) were performed in 2018/2019 with single leaves, which were randomly collected from various individuals on Mt. Kleiner Isidor, stored in plastic bags on moist paper towels, transported to the laboratory, and stored in darkness at +4 °C. The transportation time from the Alpine Garden of the University of Innsbruck was 1 h, and from Mt. Kleiner Isidor it was 2 h to the lab in Innsbruck.

### 4.2. Microclimate

On Mt. Kleiner Isidor, microclimatic data were collected during the growing seasons of 2018 and 2019. Leaf temperatures and photosynthetic active photon flux density (PPFD) were recorded with a climate station (CR1000, Campbell Scientific, Logan, UT, USA). In 2018, the leaf temperature of 7 individuals was monitored with 25 thermocouples; in 2019, the temperature of 4 individuals was monitored with 12 thermocouples. In 2019, additionally the air temperature at the average canopy height (10 cm) and soil temperature at a depth of 5 cm were recorded. For the air temperature and soil temperature, assessment copper constantan thermocouples (GG-TI-28 Omega Engineering Inc., Stamford, CT, USA) were used; the air temperature sensor was protected by a solar radiation shield (RAD 06, Campbell Scientific, Logan, UT, USA). For leaf temperature records, copper constantan thermocouples (TT-TI-36, Omega Engineering Inc., Stamford, CT, USA) were attached with Transpore^TM^ (3M^TM^, Saint Paul, MN, USA) to the abaxial leaf surface. The logged data were uploaded automatically every 6 h to a server, which allowed online data monitoring. This was particularly useful after the winter, as the development of minor daily amplitudes of the soil surface temperature did forecast the loss of snow cover and hence the beginning of the growing season. Additionally, standardized air temperature data were provided by the Avalanche Warning Service Tyrol from the nearby weather station “Stubaier Gletscher-Schaufeljoch” (N 46°58.632 E 11°6.654, 3160 m a.s.l.).

### 4.3. Detection of In Situ Freezing Exotherms

To estimate the incidence of leaf-freezing-events, the temperature data were monitored at a high frequency (0.5 s^−1^). This allowed the detection of leaf-freezing-events, as, by the phase transition from liquid water to ice, crystallization heat is released, so-called freezing exotherms. A characteristic and sudden leaf temperature increase, which was not detectable in the air temperature, indicated the freezing exotherms. Exotherms were detected with computer aid by searching leaf temperature increases $\Delta T\;=\;T_{\left(t\right)}-T_{\left(t-10s\right)}$ within 10 s. This is a modification of the so-called Differential Thermal Analysis (DTA), which is an established method [51], but mainly applied under controlled laboratory conditions. The ice nucleation temperatures presented are the temperatures immediately before the leaf temperature increase.

### 4.4. Leaf Freezing Effects and Assessment of Mechanistic Behavior

All the following experiments were performed in the lab. Generally, cold-temperature scenarios were simulated inside cooling compartments of fully temperature controlled commercial chest freezers (for details see Neuner, Huber, Plangger, Pohlin and Walde [11]). With this freezing system, it is possible to control the treatment temperature with very low temperature fluctuations (<0.2 °C). Moreover, any desired temperature (e.g., leaf temperature) can be monitored by a set of 24 copper constantan thermocouples (TT-TI-36, Omega Engineering Inc.). In 2018 and 2019, the cooling and thawing rates were limited to a maximum of 3 °C h^−1^. In the experiments of 2011, due to limitations of the cooling system of the confocal microscope the cooling rates were 10.8 °C h^−1^.

#### 4.4.1. Determination of Freezing Resistance

The freezing resistance of leaves of *R. glacialis* was assessed by the simulation of artificial frost nights with different severities in the temperature-controlled commercial chest freezers. The experimental protocol is described precisely but briefly: Leaves were laid out on wet paper towels inside of a sealable plastic bag. To avoid the artificial supercooling of the samples, ice nucleation was triggered by the application of a few droplets of an ice nucleation active (INA) bacteria suspension (*Pseudomonas syringae* van HALL 1902) to the wet paper towels. For each treatment temperature, 10 randomly selected leaves were chosen. Treatment temperatures were set in a sequence with at maximum 3 °C difference. After controlled cooling (−3 °C h^−1^) to the target temperature, the leaves were exposed to the target temperature for 4 h, followed by controlled thawing (+3 °C h^−1^). After the treatment, the samples were stored at +20 °C under moderate light conditions until the frost damage became visible. Damage was assessed by the chlorophyll fluorescence. The chlorophyll fluorescence (Fv/Fm) was measured either with the MINI PAM (Walz, Effeltrich, Germany) or the IMAGING-PAM M-Series (MAXI Version, Walz).

$LT_{50}$ is the temperature at which 50% of the tested leaves are considered damaged. For the calculation of $LT_{50},$ a logistic function (Boltzmann function) was fitted to the data: $Fv/Fm=\frac{{\left(\frac{Fv}{Fm}\right)}_{min}-{\left(\frac{Fv}{Fm}\right)}_{max}}{1+e^{\frac{T-T_{LT50}}{dx}}}+{\left(\frac{Fv}{Fm}\right)}_{max}$, where T is the temperature, $\frac{Fv}{Fm}$ is photosynthetic yield or viability in %, ${\left(\frac{Fv}{Fm}\right)}_{min}$ and ${\left(\frac{Fv}{Fm}\right)}_{max}$ are the asymptotic lower and upper limits of the curve (minimum yield, maximum yield), $T_{LT50}$ is the temperature at the inflection point, and dx is a slope factor. After randomly selecting 4 yield values from each target temperature, a fit analysis was performed. This procedure was repeated 250 times. The mean LT_50_ values and standard deviation are presented.

#### 4.4.2. Infrared Differential Thermal Analysis

For spatio-temporal analysis of the freezing behavior of *R. glacialis* individuals, potted plants were monitored during freezing treatments down to −14 °C by Infrared Differential Thermal Analysis (IDTA). Infrared Differential Thermal Analysis spots ice nucleation, monitors ice propagation, and detects also supercooling. For IDTA, a digital infrared camera (ThermaCAM S60, FLIR Systems, Danderyd, Sweden) was placed on a custom-made lid on top of a commercial chest freezer. The lid has an aperture for the optic of the infrared camera, which allowed monitoring the samples throughout the freezing treatment. The digital infrared camera recorded images at a frequency of 15 frames s^−1^. The IDTA subtraction of previous images was processed by Researcher Pro (version 2.10, FLIR Systems), as described previously in detail [52].

#### 4.4.3. Gas Exchange

To test whether the ice nucleation in leaves alters the leaf photosynthetic performance, gas exchange measurements were performed before, one hour after, and one day after a controlled freezing temperature treatment.

Gas exchange measurements were started using two gas exchange measurement systems (GFS 3000, Walz) in parallel. One fully developed leaf was randomly selected and excised from each individual (*n* = 6) and placed in a vial filled with tap water. The chlorophyll fluorescence (Fv/Fm) and gas exchange parameters (A: net assimilation rate; R_d_: dark respiration rate; E: transpiration rate: GH2O diffusive conductance) were continuously recorded during a period of 1 h (PPFD: 1500 µmol photons m^−2^·s^−1^; T_leaf_: 20 °C; flow: 750 µmol·s^−1^; CO_2_: 400 ppm; H_2_O: 15,000 ppm), to allow the gas exchange parameters to come into a steady state.

Fine wire thermocouple sensors (TT-TI-36, Omega Engineering Inc.) were mounted to the leaf blade by adhesive tape Transpore^TM^ (3M^TM^, Saint Paul, MN, USA) to control the leaf temperature during the following exposure to freezing temperatures. Two different experimental approaches were followed:

(1) Unfrozen leaves: Six leaves were cooled at a rate of −3 °C h^−1^ down to −1 °C. After a 4 h exposure to −1 °C, the leaves were rewarmed (+5 °C h^−1^) to +4 °C until further use.
(2) Frozen leaves: Six leaves were cooled at a rate of −3 °C h^−1^ down to −3 °C. After a 4 h lasting exposure to −3 °C, the leaves were rewarmed (+5 °C h^−1^) to +4 °C until further use. To ensure ice nucleation and extracellular ice formation in the leaf mesophyll, INA-bacteria (ice nucleation temperature approx. −2.3 °C) were added to the water one day before the freezing treatment.

Gas exchange measurements of each individual leaf from both experimental approaches were determined 1 h and 1 d after the end of the freezing exposure treatments. To minimize the effect of different stomatal opening, the net assimilation rate A as well as the dark respiration rate R_d_ were divided by the diffusive conductance GH2O.

#### 4.4.4. Cryo-Microscopy in Reflected Polarized Light

How ice is accommodated in viable leaves at sublethal temperatures was assessed by the recently developed method: cryo-microscopy in reflected polarized light (CM_rpl_; [23]). By CM_rpl_, it is possible to visualize and localize ice crystals unambiguously in plant tissues. Briefly, a reflected light microscope (BXFM-F, Olympus, Tokyo, Japan) was placed in a fully temperature-controlled environment. Inside the controlled environment, entire leaves were frozen to sublethal temperatures; ice nucleation was ensured by INA bacteria. At the target temperature (−7 °C), leaves were sectioned transversally with a razorblade and the cut surface was immediately inspected with the CM_rpl_. With crossed polarization filters, the visibility of ice is enhanced, while the liquid water and surface reflections disappear.

#### 4.4.5. Cryo-Microscopy

The dynamic and extent of freezing cytorrhysis of mesophyll cells of *R. glacialis* was studied on leaf cross sections exposed to a controlled freezing treatment under the microscope. Temperature control was performed by a specifically developed temperature-controlled chamber for the stage of an inverted light microscope (light microscope temperature-controlled chamber, LM-TCC; [18]). Cross sections were cooled at a maximum rate of 10.8 °C h^−1^ down to a sublethal temperature of −8 °C.

The cellular reactions throughout the freezing treatment were monitored by a Zeiss Axiovert 200M inverted microscope; microscope control was provided by the software AxioVision Rel. 4.7, and for image acquisition an Axiocam Mrc5 camera was used (all the components and software from Carl Zeiss, Jena, Germany). For plasma membrane staining, the amphiphilic fluorescent styryl dye FM 1–43 was applied. Cross sections were exposed to a 20 µM Film Tracer FM 1–43 (green biofilm cell stain, Invitrogen Ltd., prepared from a 20 mM stock solution in DMSO) for 10 to 15 min [53].

To investigate the changes in the plasma membrane, improve the visibility of individual cells, and facilitate the tracking of changes in cell size, confocal laser scanning microscopy (CLSM) was used with LSM 5 PASCAL (Carl Zeiss). Samples were excited by an argon laser beam (488 nm). Emission was collected by two separate channels: (1) band-pass filter (false colored green) at 505–550 nm, and (2) long-pass filter (false colored red) at 560 nm. For details regarding confocal laser scanning microscopy, see Holzinger, Lütz and Karsten [53].

The acquired image series in the course of the freezing treatments were analyzed as follows: As the 3D-shape of spongy parenchyma cells is very heterogeneous, we therefore opted for 2D analysis and measured the maximum cell area (CA) at the middle focal plane by outlining cells with the freehand selection tool in ImageJ [54]. For each individual cell, the measured CA at a certain freezing temperature was related to the non-frozen (+20 °C) one, so that the share of the CA could be calculated. For the palisade cells, the procedure was the same, but, additionally, as the palisade cells follow a cylindrical shape, the dimensions of the cell diameter and the cell length were assessed. By observing the changes in diameter and length, we aimed to elucidate whether palisade cells respond biased to extracellular ice formation.

#### 4.4.6. Raman Imaging

Raman imaging was used to map the chemical composition of *R. glacialis* leaves in context with the microstructure and a spatial resolution of 0.4 µm [55]. Razor blade-cut microsections were treated with EtOH (to reduce fluorescence); placed on a glass slide with a drop of D2O; and closed with a cover slip, which was sealed with nail polish. Using a Confocal Raman microscope (Alpha300RA, WITec GmbH, Germany) equipped with a 532 nm laser line, a spectrometer (600 g mm^−1^ grating, UHTS 300, WITec GmbH, Germany), a CCD camera (DV401A-BV-351, Andor, Belfast, Northern Ireland) and a 100× oil immersion objective (numerical aperture = 1.4, coverslip corrected 0.17 mm) (Carl Zeiss), hyperspectral maps were acquired of the regions of interest. In some cases, the section quality was good enough to map across the entire leaf section (Figure 6). Raman scans were recorded with a step size 0.33 µm and an integration time of 0.43 s/pixel. Raman images were generated after cosmic ray removal and baseline correction and are based on band integration and non-negative matrix factorization (NMF) after baseline correction [55].

### 4.5. Data Analysis and Statistics

All the data processing and analysis, including the statistical and regression analyses, were performed with R [56]. A significance level of 5% was selected. The difference in the reduction in the CA area between spongy cells and palisade cells was tested by the Wilcoxon Rank Sum Test. Gas exchange data were analyzed by “two-way repeated measures ANOVA” with the package “Rstatix” [57]. Normality was tested by residual diagnostics.

## 5. Conclusions

At our investigation site in the nival zone, atmospheric temperatures insufficiently describe the environmental strain of leaves of *R. glacialis*. Severe nocturnal leaf temperature depressions occurred, whereas the daily maximum temperature greatly exceeded the atmospheric temperatures. Hence, atmospheric temperatures may not be used to assess plant performance and distribution patterns or for modelling at such elevations. *Ranunculus glacialis* is outstanding in maintaining a high degree of freezing resistance during the reproduction growth period. During our investigations, the leaf temperatures never reached the lethal temperatures. However, *R. glacialis* is frequently exposed to subzero temperatures. In a first line of defense, the leaves remained unfrozen until ice formation happened (−2.6 ± 0.9 °C) and spread through the mesophyll. Indeed, ice from a single leaf did not necessarily spread into the other leaves, suggesting the soil acting as a thermal ice barrier. During the second line of defense, after ice nucleation, ice is accommodated in the intercellular spaces of the spongy parenchyma and all mesophyll cells undergo severe, reversible freezing cytorrhysis. No photosynthetic limitations after ice formation could be observed. The mesophyll is characterized by huge intercellular spaces and a high cell wall flexibility. The pectin richness may be responsible for the high flexibility and water permeability during the freeze dehydration of cells. The mapping of triglyceride in the spongy parenchyma, where the majority of ice masses are located during freezing, may be the starting point for future research.

## Supplementary Materials

The following are available online at https://www.mdpi.com/1422-0067/21/19/7042/s1. Figure S1. IDTA images obtained on a potted *R. glacialis* plant during controlled freezing. White coloring indicates increased temperature caused by heat release during the freezing of water. The time series starts 65 min after the first leaf of the plant froze (a–g). *R. glacialis* leaves froze one after another. Ice rapidly spread from the starting points into the whole leaf blade. The green circles indicate from which location the temperature is displayed in the bottom left. Elapsed time since the first freezing event was detected is indicated in the bottom right corner. Figure S2. IDTA video sequence of a single leaf of *R. glacialis* freezing. White coloring indicates increased temperature caused by heat release during the freezing of water.Figure S3. Fit of Raman endmember spectra with the reference spectra: (a) Pectin-rich endmember (Figure 6f, red spectrum) shows the best coincidence with a pure pectin (55–70%) esterified (blue spectrum). (b) The cell wall (Figure 6f, blue spectrum) was best fit with three components and the major cellulose composition was confirmed. (c) The lipid endmember (Figure 6f, green spectrum) shows the same marker bands with tripalmitin.

## Abbreviations

A	Net assimilation rate
A/GH2O	Net assimilation rate divided by the diffusive conductance
CA	Cell area
CLSM	Confocal laser scanning microscopy
CM_rpl_	Cryo-microscopy in reflected polarized light
D2O	Deuterium oxide
DMSO	Dimethyl sulfoxide
DTA	Differential thermal analysis
EtOH	Ethanol
FIVs	Freeze induced vesicles
GH2O	Diffusive conductance
IDTA	Infrared differential thermal analysis
INA	Ice nucleation active
LM-TCC	Light microscope-temperature-controlled chamber
LT_50_	Median lethal temperature
NMF	Non-negative matric factorization
PPFD	Photosynthetic active photon flux density
R_d_	Dark respiration rate
R_d_/GH2O	Dark respiration rate divided by the diffusive conductance
ROS	Reactive oxygen species
