# Supplementary figures and images for: Winter Nights during Summer Time: Stress Physiological Response to Ice and the Facilitation of Freezing Cytorrhysis by Elastic Cell Wall Components in the Leaves of a Nival Species
Source: Int J Mol Sci. 2020 Sep 24;21(19):7042. doi: 10.3390/ijms21197042 (PMC7582304; doi:10.3390/ijms21197042)

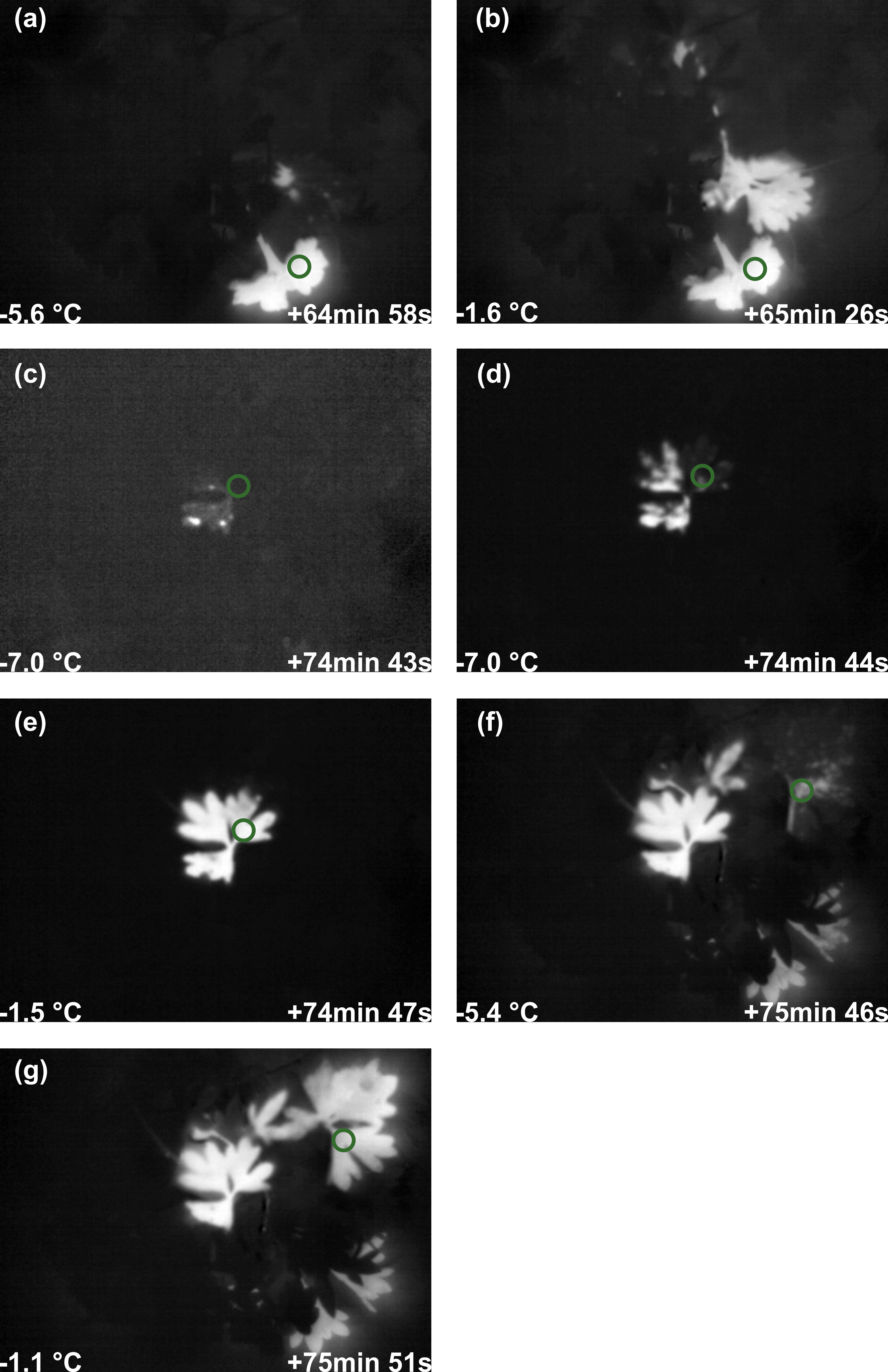

Supplement: Supplementary file 1 [file ijms-21-07042-s001.zip › figS1_IDTA_supp.tif]

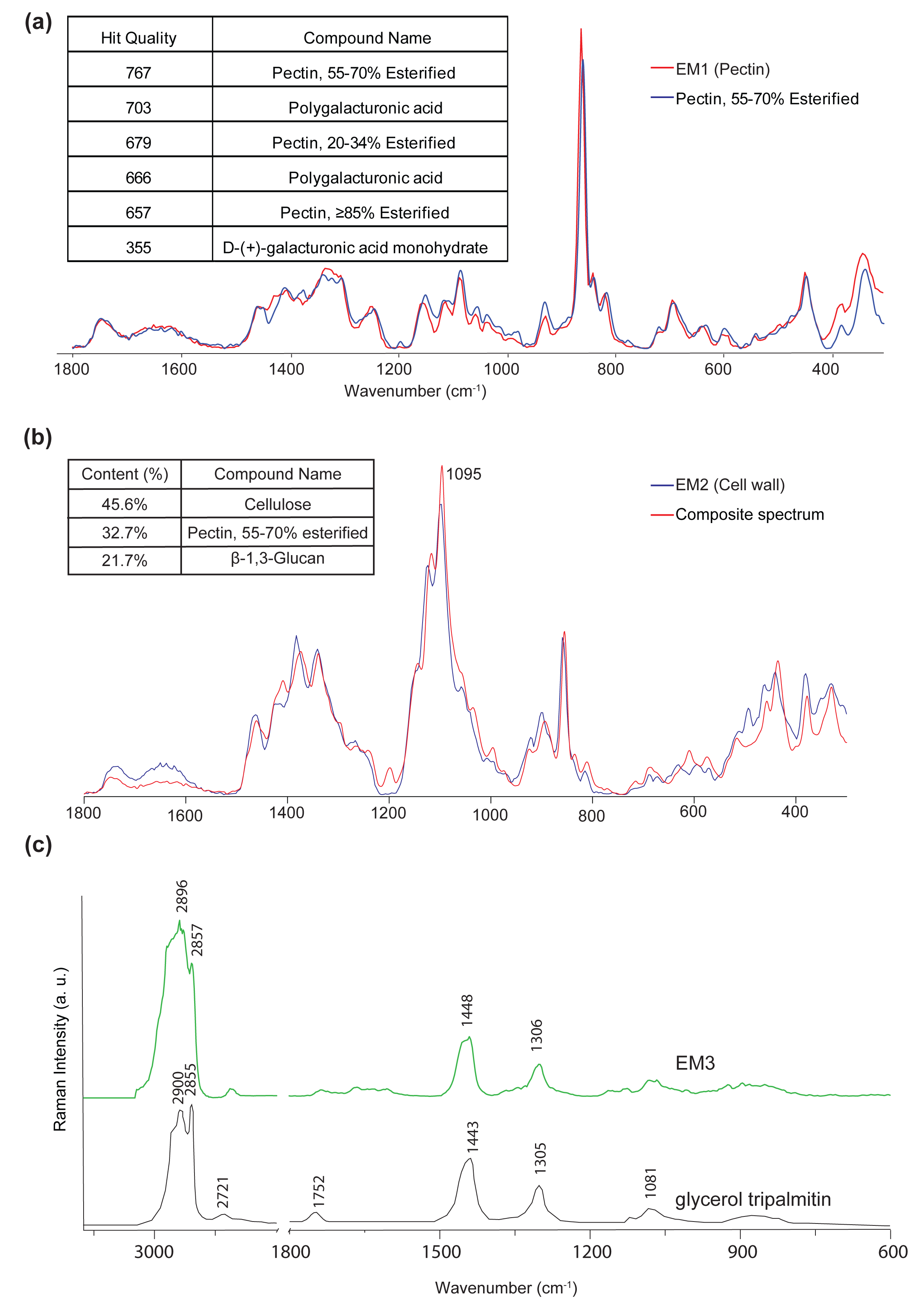

Supplement: Supplementary file 1 [file ijms-21-07042-s001.zip › figS3_raman_supp_revision.tif]
